# Exacerbation of blast-induced ocular trauma by an immune response

**DOI:** 10.1186/s12974-014-0192-5

**Published:** 2014-11-29

**Authors:** Courtney Bricker-Anthony, Jessica Hines-Beard, Lauren D’Surney, Tonia S Rex

**Affiliations:** 1 Vanderbilt Eye Institute, Vanderbilt University, 11425 MRB IV, 2213 Garland Ave., Nashville, TN 37232 USA; 2 Vanderbilt Brain Institute, Vanderbilt University, 11425 MRB IV, 2213 Garland Ave., Nashville, TN 37232 USA; 3 Department of Ophthalmology, University of Tennessee Health Science Center, 930 Madison Ave., Memphis, TN 38103 USA

**Keywords:** Eye trauma, Immune response, Vision loss, Cell death, Neurodegeneration

## Abstract

**Background:**

Visual prognosis after an open globe injury is typically worse than after a closed globe injury due, in part, to the immune response that ensues following open globe trauma. There is a need for an animal model of open globe injury in order to investigate mechanisms of vision loss and test potential therapeutics.

**Methods:**

The left eyes of DBA/2 J mice were exposed to an overpressure airwave blast. This strain lacks a fully functional ocular immune privilege, so even though the blast wave does not rupture the globe, immune infiltrate and neuroinflammation occurs as it would in an open globe injury. For the first month after blast wave exposure, the gross pathology, intraocular pressure, visual function, and retinal integrity of the blast-exposed eyes were monitored. Eyes were collected at three, seven, and 28 days to study the histology of the cornea, retina, and optic nerve, and perform immunohistochemical labeling with markers of cell death, oxidative stress, and inflammation.

**Results:**

The overpressure airwave caused anterior injuries including corneal edema, neovascularization, and hyphema. Immune infiltrate was detected throughout the eyes after blast wave exposure. Posterior injuries included occasional retinal detachments and epiretinal membranes, large retinal pigment epithelium vacuoles, regional photoreceptor cell death, and glial reactivity. Optic nerve degeneration was evident at 28 days post-blast wave exposure. The electroretinogram (ERG) showed an early deficit in the *a* wave that recovered over time. Both visual acuity and the ERG *b* wave showed an early decrease, then a transient improvement that was followed by further decline at 28 days post-blast wave exposure.

**Conclusions:**

Ocular blast injury in the DBA/2 J mouse recapitulates damage that is characteristic of open globe injuries with the advantage of a physically intact globe that prevents complications from infection. The injury was more severe in DBA/2 J mice than in C57Bl/6 J mice, which have an intact ocular immune privilege. Early injury to the outer retina mostly recovers over time. In contrast, inner retinal dysfunction seems to drive later vision loss.

**Electronic supplementary material:**

The online version of this article (doi:10.1186/s12974-014-0192-5) contains supplementary material, which is available to authorized users.

## Background

Over 186,000 eye injuries were diagnosed in fixed (not deployed) United States military medical facilities between 2000 and 2011 [1]. These injuries were recently projected to cost the United States economy $25 billion in healthcare, work lost, and family support. In addition, each year approximately 50,000 United States citizens experience permanent vision loss as a result of trauma [2-4]. While most civilian traumatic eye injuries are unilateral and due to blunt force trauma, a large proportion are bilateral in the military population and are caused primarily by blast wave exposure [5].

Open globe injury refers to an insult that perforates or penetrates the eye, accounting for approximately 40% of all ocular injuries in military service members [5,6]. Visual outcomes are worse after open globe trauma as compared to closed globe due to the increased incidence of ocular inflammation (such as endophthalmitis) that can lead to greater corneal damage, and proliferative vitreoretinopathy [7]. This is in addition to common pathologies shared between open and closed globe trauma, including retinal tears, retinal detachments, choroidal ruptures, and optic nerve atrophy [7].

Treatment for eye trauma has been impeded by the lack of suitable animal models that recapitulate the initial injury. We have developed an experimental system that mimics the primary blast wave experienced by military service members [8]. This system directs a blast of overpressure air at the mouse eye. When directed at the eyes of C57Bl/6 J mice, the blast induces subtle changes during the first weeks after injury, with significant visual deficits developing over time [9].

In this study we use our eye-directed blast model on a mouse that lacks a molecularly intact blood ocular barrier. The DBA/2 J mouse lacks C5 and CD94, necessary components of the anterior chamber-associated immune deviation (ACAID), which controls ocular immune privilege in the anterior part of the eye [10]. Our goal was to generate a model of open globe trauma by inducing open globe symptoms while retaining a physically intact eye, thus allowing for longitudinal study of injury progression without complications due to infection. We have characterized the effects of blast waves on the cornea, retina, optic nerve, and visual function during the first month post-blast wave exposure.

## Methods

### Animals

Three-month-old DBA/2 J (n = 85), were used in this study (The Jackson Laboratory, Bar Harbor, Maine, United States). Mice were maintained on a 12 hour light/dark cycle and provided access to food and water *ad libitum*. All experimental procedures were approved by the Institutional Animal Care and Use Committee of Vanderbilt University (protocol # M/12/132), according to the Association for Assessment and Accreditation of Laboratory Animal Care guidelines. The DBA/2 J mouse is susceptible to developing glaucoma from about six months of age; therefore, all mice were collected at four months of age to avoid glaucoma-related complications [11]. These mice have reactive microglia in the retina at three months of age, indicating a heightened neuroinflammatory state even in the absence of trauma [12]. Therefore, age-matched controls were used throughout the study.

### Ocular blast injury

Blast wave exposure was performed as previously described [8]. Briefly, anesthetized mice were secured and padded within a housing chamber that was slid within a larger tube composed of polyvinylchloride (PVC), which shielded the body and head of the mouse from the blast wave. The outer PVC tube had a machined hole corresponding to the size of a mouse eye. The left eye of the mouse was positioned against a hole in the tube and was aligned with the barrel of the blast device. An overpressure airwave was produced by a modified paintball marker (Empire Paintball, Sewell, New Jersey, United States). Experiments were performed at blast pressures of 23, 26, or 30 psi.

### Anterior injuries

Gross pathology was assessed prior to injury and at three, seven, 14, and 28 days following blast wave exposure using an SZX16 stereomicroscope (Olympus, Center Valley, Pennsylvania, United States). Representative images were taken using a DP71 camera (Olympus, Center Valley, Pennsylvania, United States). Eyes were examined for the presence of corneal abrasions (CA), corneal edema (CE), calcification, corneal growths, blood in the anterior chamber (hyphema), cataracts, corneal neovascularization (CNV), torn irides/pupilomotor deficit (PMD), optic nerve avulsion, lid edema, and torn extraocular muscle tissue.

### Ultra-high resolution optical coherence tomography

The eyes of mice were dilated with 1% tropicamide. Systane Ultra drops (Alcon, Fort Worth, Texas, United States) were used to keep the eyes moist. The mice were wrapped in gauze, placed in a holding chamber, and head position was stabilized with a bite bar. A Bioptigen ultra-high resolution spectral domain optical coherence tomography (OCT) system with a mouse retinal bore (Bioptigen, North Carolina, United States) was used to image the retinas. Measurements were made using digital calipers in the Bioptigen software.

### Visual acuity

The Optomotry optokinetic nystagmus (OKN) system (Cerebral Mechanics, Lethbridge, Alberta, Canada) was used to assess photopic visual acuity in awake mice (n = 9) at three, seven, 14, and 28 days post-blast wave exposure. A step-wise, masked protocol was used. Mice were acclimated to the testing chamber for five minutes prior to the initiation of each test. Spatial frequency for visual acuity was 0.042 c/d.

### Flash electroretinogram

A Diagnosys Espion electrophysiology system (Lowell, Massachusetts, United States) with heated mouse platform was used to perform flash electroretinograms (ERGs) at seven, 14, and 28 days post-blast wave exposure. Dark-adapted mice (n = 14) were anesthetized with a ketamine/xylazine cocktail (Ketaset, Pfizer, New York, New York, United States; AnaSed, Lloyd, Inc., Shanandoah, Iowa, United States) and eyes were dilated with a 1% tropicamide solution. Mice were exposed to flashes of light ranging from −2 to 2.88 log cd*s/m^2^ with a flash frequency of 2,000 Hz. For flashes below −1 log cd*s/m^2^, the inter sweep delay was 10 seconds, for the −1 log cd*s/m^2^ flash it was 15 seconds, and for all remaining flashes the delay was 20 seconds. Oscillatory potentials were measured at 3 log cd*s/m^2^ sampled at 2,000 Hz with an inter sweep delay of 15 seconds. Amplitudes were measured from baseline to peak.

### Tissue collection

Mice were perfused with 4% paraformaldehyde (PFA; Electron Microscopy Sciences, Hatfield, Pennsylvania, United States) and phosphate buffered saline (PBS). Following the perfusion, the eyes and optic nerves were placed in either 4% PFA (for immunohistochemistry) or 4% PFA with 0.5% glutaraldehyde (for resin).

### Eye histology

For histological analysis, eyes (n = 20) from mice that did not receive eye drops after blast wave exposure were post-fixed, bisected, embedded in resin, sectioned on a microtome, and stained with toluidine blue (Fisher, Waltham, Massachusetts, United States). Representative images were collected on an Olympus Provis AX70 (Olympus, Center Valley, Pennsylvania, United States) with a 60x oil objective lens. To quantify retinal pigment epithelium (RPE) damage, a grading scale was developed to classify the vacuoles: 1 (normal, very infrequent and small), 2 (small and infrequent), 3 (small and frequent), 4 (large and infrequent), and 5 (large and frequent). The number of pyknotic nuclei in the outer nuclear layer (ONL) or inner nuclear layer (INL) was quantified within a single section of retina through the middle of each eye.

For immunohistochemistry, eyes (n = 65) were cryoprotected in 30% sucrose overnight at 4°C, embedded in Tissue Freezing Medium (Triangle Biomedical, Durham, North Carolina, United States) and then sectioned on a cryostat (Fisher, Pittsburgh, Pennsylvania, United States). Sections of 10-μm thickness were collected in round on 12 slides, such that each slide contained representative sections from the entire eye.

### Optic nerve histology

Optic nerves were post-fixed in 1% osmium tetroxide in 0.1 M cacodylate buffer, dehydrated in a graded ethanol series and embedded in Spurr’s resin (Electron Microscopy Sciences, Hatfield, Pennsylvania, United States). Sections of 1-μm thickness were collected on a Reichert-Jung Ultracut E microtome (Leica Microsystems, Vienna, Austria) and stained with 1% p-phenylenediamine in 50% methanol (Sigma-Aldrich, St Louis, Missouri, United States). Optic nerve sections were examined for the presence of degenerating axons on an Olympus Provis AX70 microscope using a 100x oil immersion objective lens.

### Retina immunohistochemistry

Slides were rinsed with PBS (Sodium chloride [SX0420-3, Millipore, Darmstadt, Germany], potassium chloride [P3911-500, Sigma-Aldrich, St. Louis, Missouri], sodium phosphate dibasic [S374-500, Fisher, Pittsburgh, Pennsylvania, United States] and potassium phosphate monobasic [P285-500, Fisher, Pittsburgh, Pennsylvania, United States]) and incubated at room temperature in normal donkey serum (530–100, Millipore) at 1:20 in 0.1 M phosphate buffer (Sodium phosphate monobasic [BP329-500, Fisher, Pittsburgh, Pennsylvania, United States] and sodium phosphate dibasic [S374-500, Fisher, Pittsburgh, Pennsylvania, United States]) with 0.5% bovine serum albumin (BP1600-100, Fisher, Pittsburgh, Pennsylvania, United States) and 0.1% Triton X 100 (H5142, Promega, Madison, Wisconsin, United States) (PBT) for two hours. The slides were incubated overnight at 4°C in primary antibody in PBT (Table 1), rinsed with PBS and incubated with a secondary antibody (Life Technologies, Grand Island, New York, United States) for two hours at room temperature. Slides were rinsed with PBS and mounted in Vectashield Mounting medium with 4’,6-diamidino-2-phenylindole (DAPI; Vector Laboratories, Burlingame, California, United States) for imaging on a Nikon Eclipse epifluorescence microscope (Nikon, Melville, New York, United States) or Olympus FV-1000 confocal microscope (Olympus, Center Valley, Pennsylvania, United States). Imaging on the Olympus FV-1000 microscope was performed through use of the Vanderbilt University Medical Center Cell Imaging Shared Resource.

**Table 1:** **Antibodies used in this study**

**Antigen**	**Dilution**	**Manufacturer**	**Catalog number**
Caspase-1	1:100	Millipore, Billerica, MA	AB1871
Caspase-3	1:10	Abcam, Cambridge, MA	ab4051
Choline acetyltransferase	1:25	Abcam, Cambridge, MA	ab34419
Complent 3d	1:100	Abcam, Cambridge, MA	ab15881
GFAP	1:400	Dako, Carpinteria, CA	Z0334
Iba1	1:500	Wako, Richmond, VA	019-19741
Nitrotyrosine	1:500	Millipore, Billerica, MA	06-284
RIP1	1:100	Santa Cruz, Santa Cruz, CA	sc-7881
RIP3	1:100	Santa Cruz, Santa Cruz, CA	sc-47364

GFAP, glial fibrillary acidic protein; Iba1, ionized calcium-binding adapter 1; RIP1, receptor-interacting protein kinase 1; RIP3, receptor-interacting protein kinase 3.

### Tdt dUTP nick end labeling quantification

Eye sections from mice treated with non-medicated eye drops were labeled with the Tdt dUTP nick end labeling (TUNEL) Apoptosis Detection Kit adhering to the manufacturer’s protocol (Merck Millipore, Darmstadt, Germany) and mounted with Vectashield Mounting Medium with DAPI. TUNEL-positive cells within the ONL, INL and ganglion cell layer (GCL) were counted and the lengths of the regions with TUNEL-positive cells (affected regions) were measured using NIS Elements Advanced Research software (Nikon, Melville, New York, United States). The total length of each retinal section with TUNEL-positive cells (affected section) was also measured. In order to determine the percentage of the retina with cell death, we measured and summed the lengths of all sections on the slide. Then, we divided the sum of affected region lengths by the total length of all sections and multiplied this value by 100, which yielded the percentage of retina with TUNEL-positive cells.

### Statistical analysis

All statistical analyses were calculated using Graphpad Prism software (San Diego, California, United States). A one-way ANOVA with a Bonferroni *post-hoc* test was used to analyze ERG and visual acuity data. The means ± SEM were calculated and presented for each data set.

## Results

### Ocular trauma induces corneal and lens damage

**Figure 1:**
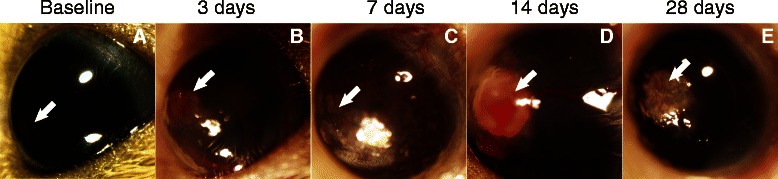
**Blast trauma injures the ocular surface. (A)** The majority of mice had calcium deposits in the cornea at baseline. Images showing the most common anterior pathologies detected at three, seven, 14, and 28 days post-blast wave exposure. **(B)** Corneal edema and hyphema at three days post-blast wave exposure. **(C)** Corneal edema and neovascularization at seven days post-blast wave exposure. **(D)** A corneal growth with neovascularization and hyphema at 14 days post-blast wave exposure. **(E)** Corneal scarring and neovascularization at 28 days post-blast wave exposure. Arrows indicate pathologies.

Blast wave exposure caused numerous anterior injuries that, in some cases, varied depending on time after the blast (Tables 2, 3, and 4). This is in contrast to the lack of anterior pathologies in the majority of C57Bl/6 mice after eye blast [8,9]. In both cases, no eye drops or ointments were provided in order to detect all pathologies caused by an eye-directed blast to the naïve eye. Representative images of these pathologies after exposure to a 26 psi blast wave are shown in Figure 1. The eyes appeared normal immediately after the blast wave, but at three days significant pathologies were present including CE, hyphema, cataracts, and a few cases of CNV. The incidence of CE after a 23, 26, or 30 psi blast wave remained high up to 28 days; 100%, 86%, and 60%, respectively. The incidence of hyphema in all blast groups peaked at three days after blast wave exposure and was completely absent at 28 days post-blast wave exposure. The percentage of eyes with hyphema at three days was 8%, 26%, and 25% after a 23, 26, and 30 psi blast wave, respectively. In contrast, the number of eyes with CNV increased over time post-blast wave exposure. At three days post-blast wave exposure 31%, 17%, and 0% of 23, 26, and 30 psi eyes, respectively, exhibited signs of CNV. At 28 days 80%, 29%, and 45% of 23, 26, and 30 psi eyes, respectively, had CNV. Exposure to a 26 psi blast wave induced the most reproducible and clinically relevant damage profile. Therefore, this pressure level was used for the remaining experiments.

**Table 2:** **Gross pathology after a 23 psi blast wave**

**Type of injury**	**0 day**	**3 days**	**7 days**	**14 days**	**28 days**
	**(15)** ^**a**^	**13**	**13**	**8**	**5**
Corneal abrasion	2 (14)^b^	0	0	0	1 (20)
CE	0	9 (69)	3 (23)	4 (50)	5 (100)
CNV	0	4 (31)	4 (31)	5 (63)	4 (80)
Corneal scarring	0	1 (8)	3 (23)	3 (38)	2 (40)
Hyphema	0	1 (8)	0	0	0
Corneal growth	0	0	1 (8)	1 (13)	1 (20)
Torn iris	0	0	0	0	0
Traumatic cataract	0	2 (15)	3 (23)	3 (38)	3 (60)

^a^Total number of eyes examined. ^b^Number of eyes with pathology (percentage). CE, corneal edema; CNV, corneal neovascularization.

**Table 3:** **Gross pathology after a 26 psi blast wave**

**Type of injury**	**0 day**	**3 days**	**7 days**	**14 days**	**28 days**
	**(24)** ^**a**^	**23**	**14**	**9**	**7**
Corneal abrasion	4 (17)^b^	1 (4)	0	0	0
CE	0	16 (70)	5 (36)	5 (56)	6 (86)
CNV	1 (4)	4 (17)	2 (14)	4 (44)	2 (29)
Corneal scarring	0	2 (9)	1 (7)	4 (44)	5 (71)
Hyphema	0	6 (26)	1 (7)	0	0
Corneal growth	0	0	0	1 (11)	1 (14)
Torn iris	0	0	0	1 (11)	2 (29)
Traumatic cataract	0	8 (35)	1 (7)	1 (11)	1 (14)

^a^Total number of eyes examined. ^b^Number of eyes with pathology (percentage). CE, corneal edema; CNV, corneal neovascularization.

**Table 4:** **Gross pathology after a 30 psi blast wave**

**Type of injury**	**0 day**	**3 days**	**7 days**	**14 days**	**28 days**
	**(16)** ^**a**^	**16**	**14**	**11**	**11**
Corneal abrasion	3 (19)^b^	1 (6)	0	0	0
CE	0	8 (50)	9 (64)	10 (91)	8 (73)
CNV	0	0	4 (29)	6 (54)	5 (45)
Corneal scarring	0	0	0	1 (9)	0
Hyphema	1 (6)	4 (25)	2 (14)	2 (18)	0
Corneal growth	0	0	0	3 (27)	5 (45)
Torn iris	0	0	0	0	0
Traumatic cataract	0	6 (38)	6 (43)	6 (55)	7 (64)

^a^Total number of eyes examined. ^b^Number of eyes with pathology (percentage). CE, corneal edema; CNV, corneal neovascularization.

**Figure 2:**
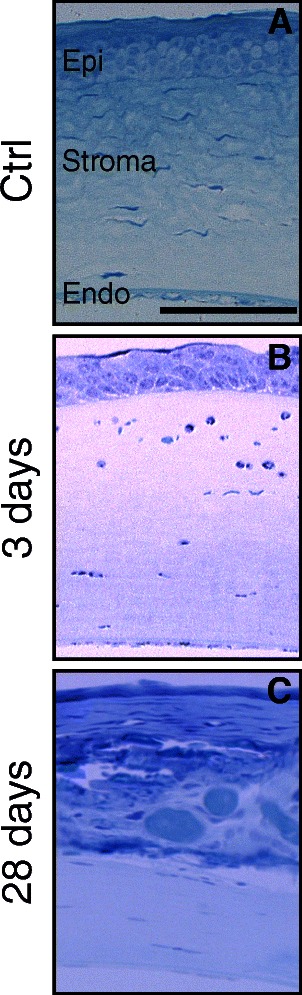
**Corneal damage persists out to 28 days post-blast wave exposure. (A-C)** Brightfield micrographs of cornea histology. **(A)** In control corneas the corneal epithelium (epi), stroma, and endothelium (endo) are well organized and show no signs of pathology. **(B)** At three days post-blast wave exposure, the epi is disorganized and the stroma contains neutrophils. **(C)** At 28 days post-blast wave exposure the epi is thin and blood vessels are evident in the stroma. The scale bar in **(A)** is 50 μm and also applies to **(B)** and **(C)**.

Consistent with the gross pathology observations, corneal damage was apparent by histology following blast wave exposure (Figure 2). Disruption of the epithelium (epi), stromal edema, and peripheral immune infiltrate were present at three days post-injury (Figure 2B). In all 28-day post-injury corneas, the epi was thin and disorganized and the stroma contained neovascularization and pockets of blood (Figure 2C).

The high frequency of corneal opacities after blast wave exposure excluded the majority of eyes from functional assessments. We hypothesized that the CE and CNV developed secondarily due to dry eye. To test this, mice were treated once with non-medicated, viscous eye drops immediately post-blast wave exposure. This prevented 90% of the corneal damage, including CE. Therefore, to perform functional (OKN, ERG) and *in vivo* anatomical (OCT) assessments, all additional mice received eye drops.

**Figure 3:**
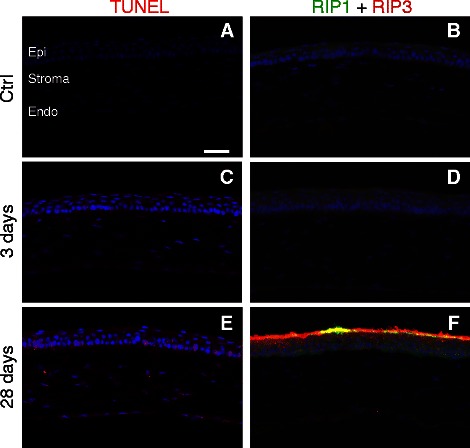
**Cell death affects each layer of the cornea and is necroptotic. (A, C, E)** Epifluorescence micrographs of TUNEL (red) and DAPI (blue). **(B, D, F)** Epifluorescence micrographs of RIP1 (green), RIP3 (red), and DAPI (blue). **(A)** In control corneas, TUNEL is rare and restricted to the epithelium (epi). **(B)** The control cornea is negative for both RIP1 and RIP3. **(C)** At three days post-blast wave exposure TUNEL is increased in the epi and is present in the stroma. **(D)** The cornea is still negative for RIP1 and RIP3 at three days post-injury. **(E)** At 28 days post-blast wave exposure, all three layers of the cornea are positive for TUNEL. **(F)** Both RIP1 and RIP3 are present in the epi, as well some light labeling in the endothelium (endo) and stroma. The scale bar in **(A)** is 50 μm and applies to all of the images in Figure 3.

Corneal cell death was examined in mice that received non-medicated eye drops. In the control cornea, occasional TUNEL-positive cells were detected in the epi as a result of normal cellular turnover (Figure 3A). At three days post-injury, TUNEL-positive cells were increased in the epi and were detected in the stroma (Figure 3C). There were no changes in immunolabeling for receptor interacting proteins 1 and 3 (RIP1, RIP3; markers of necroptosis) at three days when compared to control (Figure 3D). At 28 days post-injury, TUNEL-positive cells (Figure 3E), and increased RIP1 and RIP3 immunolabeling were detected in all layers of the cornea (Figure 3F).

### Ocular trauma causes retinal detachments

**Figure 4:**
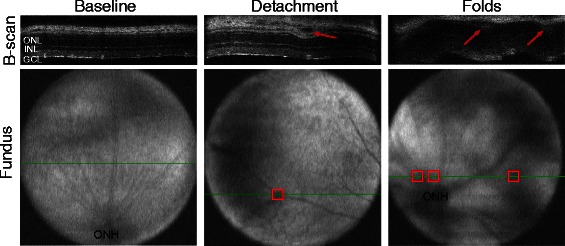
**Blast wave exposure damages the neural retina.** At baseline, each layer of the retina appears normal in the b-scan image and the corresponding fundus image shows no signs of pathology (ONH: optic nerve head). The green lines in the fundus images denote the location of the b-line scan images. An example of a retinal detachment after blast wave exposure is shown. It appears as a dark area between the retinal pigment epithelium (RPE) and photoreceptors (arrow) and as a dark shadow in the mid-peripheral region of the retina fundus image (box). An example of retinal folds with corresponding retinal detachments (arrows; boxes) is shown. ONL: outer nuclear layer, INL: inner nuclear layer, GCL: ganglion cell layer.

The majority of retinas exposed to a blast wave appeared normal at all time points by OCT imaging, despite repositioning the eye multiple times during imaging to examine all retinal quadrants (Figure 4). When retinal detachments were detected they were primarily in the mid-peripheral retina and less frequently near the optic nerve head (ONH). At three days post-blast wave exposure (n = 5), 40% of eyes had a single retinal detachment that had an average height of 0.03 mm ±0.005 (Figure 4). At seven days post-blast wave exposure (n = 10), only one eye had retinal detachments. The average height and number of the detachments at seven days was 0.05 mm ±0.03 and six per eye, respectively. One retina had a wavy appearance suggestive of epiretinal membranes and multiple retinal detachments (Figure 4). No detachments were observed at 14 days post-injury (n = 6), but it is possible that detachments were missed during imaging. At 28 days (n = 10), only the retina that appeared wavy at the seven-day time point had retinal detachments, a total of two, averaging 0.03 mm ± 0.00 in height.

### Blast wave exposure damages the neural retina, retinal pigment epithelium, and optic nerve

**Figure 5:**
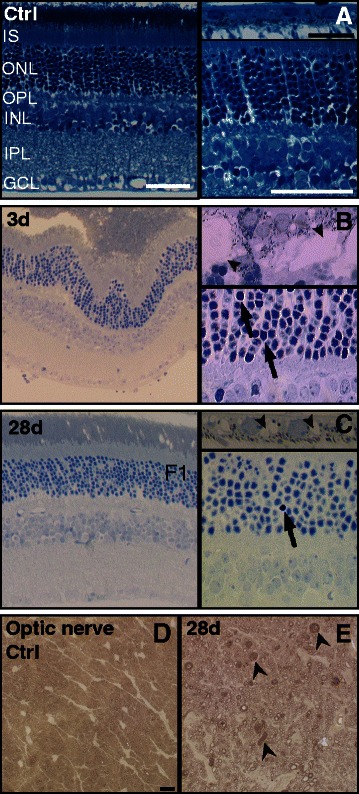
**Neuronal death, retinal pigment epithelium vacuoles and optic nerve degeneration occur after ocular trauma. (A-C)** Representative Brightfield micrographs of the retina and RPE. **(D-E)** Representative Brightfield micrographs of the optic nerve. **(A)** Control retina and RPE shows normal histology. **(B)** A retinal detachment with subretinal debris is present in conjunction with pyknotic nuclei (arrows) in the ONL. The RPE contains grade 5 vacuoles (arrowheads) and phagocytosed debris. **(C)** There are fewer pyknotic nuclei (arrow) in the retina and the RPE vacuoles (arrowheads) are smaller in size at 28 days post-blast wave exposure. **(D)** The control optic nerve appears normal. **(E)** At 28 days the optic nerve contains degenerating axons with collapsed myelin (arrowheads). The scale bars in the low and high magnification retina images are 50 μm and the scale bar in the RPE images is 20 μm. The scale bar for the optic nerve images is 5 μm. IS = inner segments; ONL = outer nuclear layer, OPL: outer plexiform layer, INL: inner nuclear layer, IPL: inner plexiform layer; GCL = ganglion cell layer; RPE = retinal pigment epithelium.

In the normal RPE, there were no vacuoles or debris accumulation present (Figure 5A). In contrast, in mice that did not receive eye drops, the RPE contained grade 5 vacuoles at three days post-blast wave exposure in the majority of eyes (67%, n = 3, Figure 5B). At 28 days after blast wave exposure, the RPE vacuoles had decreased in size to grade 2 (n = 7, Figure 5C). At both time points the RPE vacuoles were present throughout the retina. Subretinal debris, consisting of red blood cells and photoreceptor inner and outer segments, were detected in areas of retinal detachments at three days post-blast wave exposure (Figure 5B), but appeared to resolve at 28 days post-blast wave exposure (Figure 5C). RPE damage was less severe and more focal, with most of the RPE appearing normal, when mice were given non-medicated eye drops immediately after blast (Additional file 1: Figure S1).

While much of the post-blast wave retina looked normal, clusters of pyknotic nuclei were observed at three and 28 days (Figure 5B, C). The average number of pyknotic nuclei at three days post-blast wave exposure was 14 ± 10 in the ONL and 31 ± 21 in the INL. At 28 days post-blast wave exposure, the average number of pyknotic nuclei decreased to 3 ± 2 and 0 ± 0 in the ONL and INL, respectively. Optic nerves from the first week post-injury (data not shown) looked the same as those from controls (Figure 5D). In contrast, degenerating axons with collapsed myelin were prevalent at 28 days post-injury (Figure 5E).

**Figure 6:**
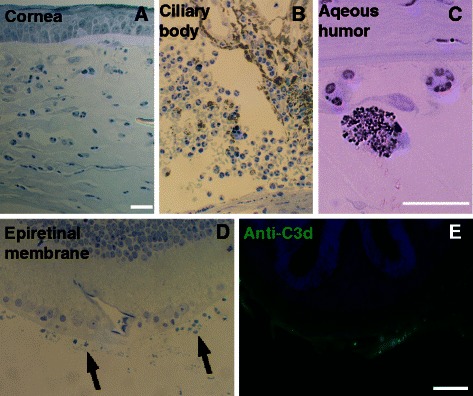
**Immune infiltrate appears after blast wave exposure. (A-C)** Immune infiltrate is present in the anterior chamber, including the cornea **(A)** and the aqueous humor near the ciliary body **(B)** and the aqueous humor near the cornea **(C). (D)** An epiretinal membrane after blast wave exposure (arrows). **(E)** The epiretinal membranes are positive for complement 3d (C3d) indicating that they are immune-mediated. The scale bar in **(A)** is 50 μm and also applies to **(B)** and **(D)**; the scale bar in **(C)** is 5 μm; the scale bar in **(E)** is 25 μm.

In eyes that did not receive eye drops, immune infiltrate was present in a subset of eyes (33%) at three days post-blast wave exposure. Infiltrate was detected in both the anterior and posterior portions of the eye, including the cornea, aqueous humor, vitreous humor, and surface of the retina (Figure 6A-C). Immune-mediated (complement 3d, C3d-positive) epiretinal membranes were occasionally detected in areas of retinal detachment (Figure 6D-E). In eyes that received non-medicated eye drops, no immune cells were detected in the anterior half of the eye, only macrophages were present in the posterior eye and only in the subretinal space, and no epiretinal membranes were detected (Additional file 2: Figure S2).

### Regional cell death occurs at multiple time points post-blast wave exposure

**Figure 7:**
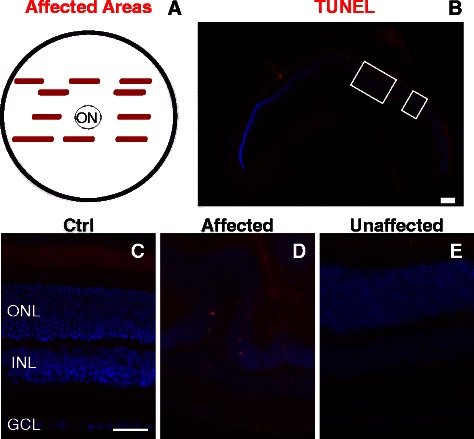
**Cell death occurs in small, focal areas after blast wave exposure. (A)** A schematic representing an enface view of the retina showing the average number and distribution of affected (TUNEL-positive) areas (red bars) detected in retinal cross-sections collected in serial through the eye three days post-blast wave exposure. **(B)** Montage of low magnification epifluorescence micrographs of a retina three days post-blast wave exposure. White boxes indicate affected areas (scale bar is 250 μm). **(C-E)** Higher magnification epifluorescence micrographs of TUNEL (red) and DAPI (blue) in a control retina **(C)**, and affected **(D)** and unaffected **(E)** regions of the retina shown in **(B)**. The scale bar in **(C)** represents 50 μm and also applies to **(D)** and **(E)**. ON = optic nerve head; GCL = ganglion cell layer; INL = inner nuclear layer; ONL = outer nuclear layer.

After blast wave exposure, all retinas had areas with TUNEL-positive cells (affected areas) at three (n = 5) and seven days post-injury (n = 9), while 82% of retinas were TUNEL-positive at 28 days post-injury (n = 11). Cell death was typically present in patches and not evenly distributed across the retina (Figure 7). These affected areas were primarily in the mid-peripheral retina, but occasionally were also detected in central retina (Figure 7A). Representative images of TUNEL in affected and unaffected areas of retina three days after blast wave exposure are shown in Figure 7D, E. Areas with retinal folds had the highest density of TUNEL-positive cells.

**Figure 8:**
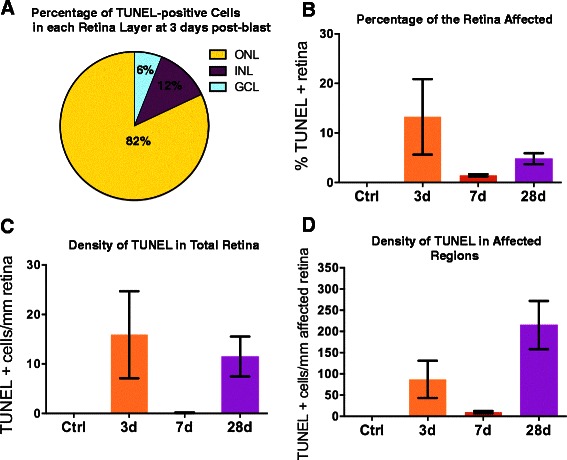
**Cell death occurs in two waves after blast wave exposure. (A)** Pie chart showing the distribution of TUNEL-positive cells through the retinal layers after blast wave exposure. **(B)** The percentage of total retina containing TUNEL-positive cells at each time point. **(C)** The average number of TUNEL-positive cells per mm total retina after blast wave exposure. **(D)** The average number of TUNEL-positive cells per mm within the affected areas after blast wave exposure. Error bars represent SEM for each time point. GCL = ganglion cell layer; INL = inner nuclear layer; ONL = outer nuclear layer.

The percentage of total retina containing TUNEL-positive cells, density of TUNEL-positive cells, and retinal layer affected were quantified (Figure 8). The majority of TUNEL-positive nuclei, 82%, were located in the ONL at three days after injury (Figure 8A). A smaller percentage of TUNEL-positive cells were detected in the INL and GCL, 12% and 6%, respectively, at three days post-blast wave exposure (Figure 8A). This ratio was similar at seven and 28 days post-blast wave exposure: 70% and 83%, respectively, located in the ONL; 27% and 16%, respectively, detected in the INL; and 3% and 1%, respectively, located in the GCL. TUNEL-positive nuclei were present in 13 ± 8% of the retina at three days post-injury, 2 ± 0.2% at seven days post-blast wave exposure, and 5 ± 1% of the retina at 28 days post-blast wave exposure (Figure 8B). When calculated in terms of total retina length, the density of TUNEL was very low, but retained the same trend of higher levels at three days as compared to 28 days. The number of TUNEL-positive nuclei per mm total retina was 15 ± 9, 0.1 ± 0.1, and 11 ± 4 at three, seven, and 28 days post-blast wave exposure (Figure 8C). Within the affected regions, the density of TUNEL-positive cells was 87 ± 44, 10 ± 3, and 215 ± 57 nuclei per mm retina at three, seven, and 28 days after blast wave exposure, respectively (Figure 8D). These results demonstrate that the area occupied by TUNEL-positive cells decreases over time, but the density of TUNEL-positive cells within affected areas increases.

### Cell death pathway markers increased after blast wave exposure

**Figure 9:**
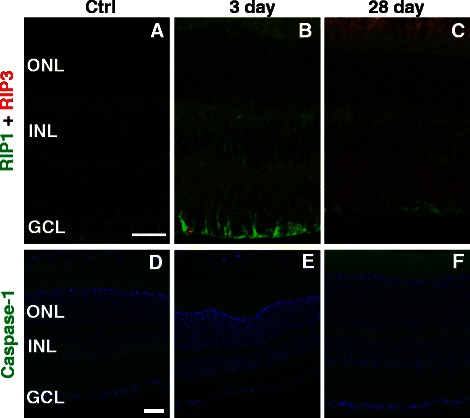
**Changes in cell death markers after blast wave exposure suggest a non-apoptotic mode of cell death. (A-C)** Confocal micrographs of retinas immunolabeled for RIP1 (green) and RIP3 (red; scale bar is 30 μm). **(D-F)** Epifluorescence micrographs of Caspase-1 (green) immunolabeling and DAPI (blue; scale bar is 25 μm). GCL = ganglion cell layer; INL = inner nuclear layer; ONL = outer nuclear layer.

Since TUNEL is a method for detecting dying cells in general (it is not specific for any cell death pathway [13]), we next used markers of apoptosis, necroptosis, and pyroptosis to gain insight into how the cells were dying. Very few caspase-3-positive nuclei were detected in the ONL at three, seven, and 28 days post-injury, even within areas of extensive cell death (data not shown). Caspase-1, a marker for pyroptosis (inflammation-mediated cell death), was present within the INL and GCL throughout the control retinas (Figure 9D). At three days post-injury (n = 3), only one third of retinas exhibited caspase-1-positive cells in the INL and GCL (Figure 9E). At 28 days after blast wave exposure (n = 5), all retinas were caspase-1-negative (Figure 9F). In the C57Bl/6 mouse caspase-1 labeling in ChAT-positive cells increased over time post-blast wave exposure [9]. Immunolabeling with anti-ChAT in DBA/2 J mice showed that these cells were still present even at 28 days post-blast wave exposure (data not shown).

In contrast, labeling for markers for necroptosis (programmed necrosis; RIP1 and RIP3) was increased after blast wave exposure. In the normal retina, RIP1 localized to the Müller glia, the inner plexiform layer (IPL), and the INL, with some light staining in the outer plexiform layer (OPL; Figure 9A). Light RIP3 staining in the normal retina was restricted to the GCL, IPL, and INL (Figure 9A). At three days post-injury (n = 4), RIP1 increased in the ONL, INL, and Müller glia, while RIP3 increased in the ONL, INL, IPL, and GCL (Figure 9B). As cell death progressed at 28 days post-blast wave exposure (n = 5), RIP1 remained elevated in the ONL and INL, while RIP3 remained elevated in the IPL and maintained some light labeling in the ONL (Figure 9C).

### Protein nitration increases in the retina after blast wave exposure

**Figure 10:**
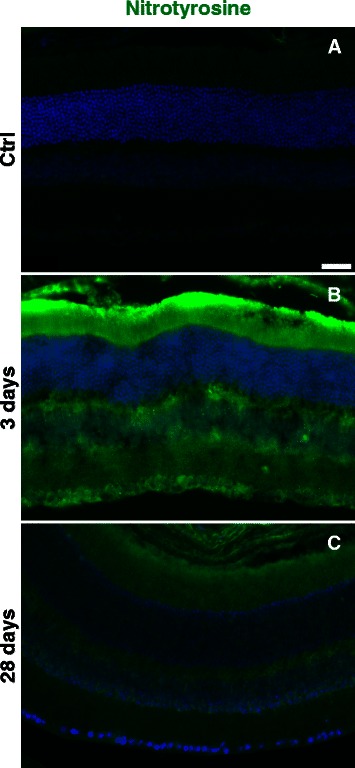
**Nitrotyrosine increases following blast. (A-C)** Representative epifluorescence micrographs of retinas immunolabeled for nitrotyrosine (green) and labeled with DAPI (blue). Scale bar is 25 μm and applies to all images.

In the control retina, nitrotyrosine immunolabeling was light and restricted to the inner retina (Figure 10A). Three days after blast wave exposure (n = 5), immunolabeling was greatly increased throughout both the inner and outer retina (Figure 10B). In eyes that received eye drops this increase was limited to focal areas (Additional file 3: Figure S3). The immunolabeling seemed less increased, but was still elevated in both the inner and outer retina at 28 days post-blast wave exposure and spread across the entire retina in eyes that received eye drops (Figure 10C, n = 5).

### Glial reactivity increases in the retina after blast wave exposure

**Figure 11:**
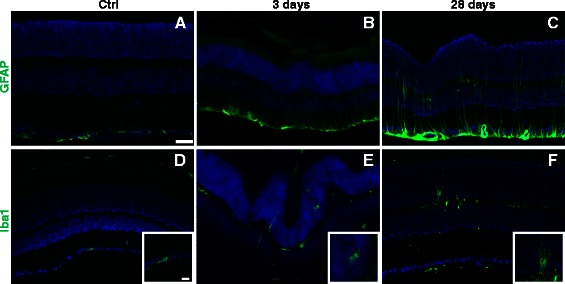
**Both Müller glia and microglia become reactive after injury. (A-C)** Representative epifluorescence micrographs of retinas immunolabeled for GFAP (green). **(D-F)** Representative epifluorescence micrographs of retinas immunolabeled for Iba-1 (green). Higher magnification images of representative microglia are present in inserts. The scale bar for the low magnification images is 25 μm and applies to all images. The scale bar for the inserts is 10 μm and applies to all images. DAPI is shown in blue.

In the normal retina, glial fibrillary acidic protein (GFAP) immunolabeling was restricted to the Müller glia end-feet and astrocytes (Figure 11A). At both three (n = 5) and 28 days (n =9) post-injury, GFAP immunolabeling was increased in the Müller cell processes (Figure 11B, C). In age-matched control retinas, immunolabeling with ionized calcium binding adaptor molecule 1 (IBA-1) showed that microglia were restricted to the inner retina and were fairly low in density (Figure 11D). Microglia were more prevalent after blast wave exposure when compared to controls beginning at three days (n =5) post-blast wave exposure (Figure 11E-F). Reactive microglia, which are amoeboid in appearance, were detected (Figure 11E-F, inserts). GFAP immunolabeling was the same in all eyes regardless of treatment or lack thereof with eye drops. However, there was less of an increase in reactive microglia in eyes that received eye drops after blast (Additional file 4: Figure S4).

### Ocular trauma results in visual deficits

**Figure 12:**
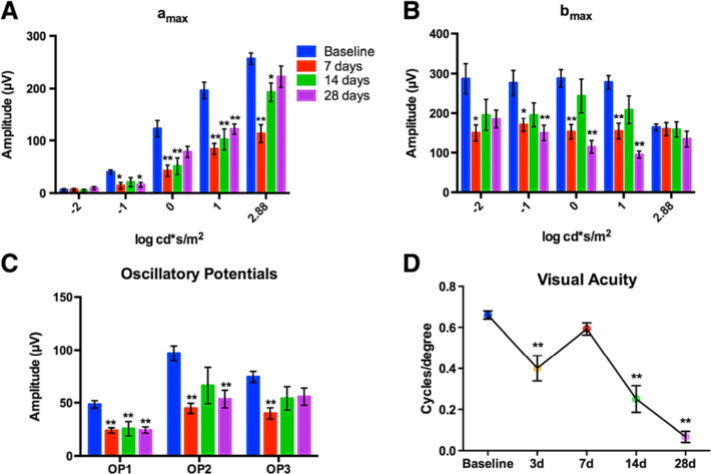
**Blast wave exposure causes early and late visual deficits. (A)** Bar graph of the electroretinogram (ERG) a_max_ over light intensity. An early decrease in the a_max_ recovers over time. **(B)** Bar graph of ERG b_max_ over light intensity. The b_max_ recovers by 14 days post-blast wave exposure then decreases again at 28 days post-blast wave exposure. **(C)** Bar graph of the oscillatory potential (OP) peak amplitude at each time point. The OPs are decreased at seven and 28 days post-blast wave exposure. **(D)** Photopic spatial threshold (visual acuity) is significantly decreased at three, 14 and 28 days post-blast wave exposure. *P <0.05, **P <0.01. Error bars represent SEM for each graph.

The ERG *a* wave amplitude (a_max_), a measure of photoreceptor function, was diminished to approximately 40% of baseline at seven days post-blast wave exposure based on an average of the responses at each light intensity (Figure 12A). At 14 days post-blast wave exposure, a statistically significant decrease is still present, but the level of decrease is less substantial at an average of 55% of baseline. At 28 days post-blast wave exposure, the a_max_ was similar to baseline at the brightest flash, but was still reduced at the other flash intensities (Figure 12A). The average deficit from baseline at the lower light intensities was 63% at 28 days post-blast wave exposure. The ERG *b* wave amplitude (b_max_), a measure of inner retinal function, was similar to baseline at the brightest light intensity at all time points post-blast wave exposure (Figure 12B). However, at all other light intensities, there was a deficit averaging 52% below baseline at seven days post-blast wave exposure, 74% of baseline at 14 days post-blast wave exposure (note: not statistically significant), and 48% of baseline at 28 days post-blast wave exposure. The decrease in the b_max_ at seven days post-blast wave exposure seems to correspond to the decrease in the a_max_ at this time point (Figure 12B). In contrast, the decrease in the b_max_, particularly at 0 and 1 log cd*s/m^2^ (*P* <0.01) at 28 days post-blast wave exposure was greater than at earlier time points despite a recovery of *a* wave. The oscillatory potentials (OP), a measure of amacrine and interneuron cell function, were also affected after blast wave exposure (Figure 12C). The OP1 was diminished at all time points after blast wave exposure (*P* <0.01). OP2 was significantly lower than baseline at both seven and 28 days (*P* <0.01). OP3 appeared lower than baseline, but only reached statistical significance at seven days post-blast wave exposure (*P* <0.01). A significant decline in visual acuity was observed at three (0.40 ± 0.06, *P* <0.01), 14 (0.25 ± 0.07, *P* <0.01), and 28 (0.07 ± 0.03, *P* <0.01) days post-blast wave exposure (Figure 12D).

## Discussion

The majority of the anterior ocular pathologies appear to be secondary to CE and dry eye (for example, CNV) as they were almost completely eliminated by treatment with lubricating, non-medicated eye drops given once, acutely, after blast wave exposure. The blast wave may disrupt the tear film, which we artificially restored with the eye drops. A recent study reported tear film deficiencies in blast-exposed veterans [14]. Preserving the corneal fluid barrier limited immune infiltrate, oxidative stress, and microglial reactivity after blast, but had no effect on macroglial reactivity, extent of cell death, or optic nerve degeneration.

A large influx of immune cells into the eye occurred after blast wave exposure in DBA/2 J mice but not in C57Bl/6 mice, which have a fully intact ACAID. The immune response after trauma elicited formation of epiretinal membranes, increased nitrosative stress, greater cell death, and more severe vision loss than was detected in the C57Bl/6 mouse [9]. Another major difference between these two strains was the acute damage to the RPE after blast wave exposure in DBA/2 J mice. Remarkably, despite the early and dramatic vacuolization and significant amount of subretinal debris present within days after blast wave exposure, the RPE and subretinal space appeared near normal at 28 days after injury. This shows the resiliency of these cells and their large capacity to phagocytose and remove debris. The differences in response to blast wave exposure by these two strains of mice is consistent with comparisons between closed globe and open globe trauma patients [7].

As in the retinas of C57Bl/6 mice post-blast wave exposure, the cell death pathway appears to be non-apoptotic in DBA/2 J mice [9]. In both strains of mice we detected increases in markers for necroptosis after blast wave exposure, suggesting that necroptosis is the main cell death pathway activated. The peak of cell death is earlier in DBA/2 J mice; three days as compared to 28 days in C57Bl/6 mice [9]. Also, more retinal ganglion cells (RGC) were TUNEL-positive in DBA/2 J mice and damage to the optic nerve was more substantial. Unlike the whole body/head blast models [15,16], photoreceptor cell death was present in this model and in a trinitrotoluene (TNT) blast study [17]. The discrepancies may be due to the physics of head movement in their model as compared to the restrained head in our model and that of Zou *et al*. [17]. Similar to Mohan *et al*., we detect RGC death and degeneration in the optic nerve [16]. They reported axon degeneration and RGC loss at 10 months post-blast wave exposure, while we show that it may begin as early as one month.

Our blast pressure is comparable to the low-level blast group exposed to TNT by Zou *et al*. [17]. Despite this, the histology and labeling for nitrosative damage and GFAP in this study is more comparable to their findings in the high-level blast exposure group at two weeks post-injury [17]. We suspect that these differences are due to the increased damage caused by immune infiltrate as blast wave-exposed C57Bl/6 mice show a phenotype more similar to their low-level blast group, as would be expected. In fact there is also evidence of immune infiltrate in their high-level blast group, but not in their low-level blast group retinas [17].

One of the most surprising results was the biphasic decrease in the flash ERG. Acutely post-blast wave exposure, the deficits in visual acuity and in the ERG amplitude appear to be driven by damage to the outer retina. The ERG amplitude decrease was photoreceptor-driven since the decrease in the a_max_ was greater than that for the b_max_. These deficits in visual function correlated with the presence of retinal detachments and extensive vacuolization and damage to the RPE in the first week after injury. The recovery of the OKN at seven days post-blast wave exposure may reflect partial resolution of the retinal detachments in combination with an improvement in the OKN response as a result of repeat testing [9,18]. A similar dip and recovery of visual function was reported by us, and others, after blast wave exposure [9,16], and is also seen in patients whose visual acuity can drop after trauma, but recover over time [5,19].

At 28 days post-blast wave exposure the profile is very different. The ERG a_max_ is improved as compared to seven days post-blast wave exposure, indicating a recovery in the outer retina. In contrast, the ERG b_max_ and spatial acuity threshold (visual acuity) continue to decrease at 28 days post-blast wave exposure. This suggests an ongoing dysfunction in the inner retina after blast wave exposure. These results are supported by the detection of a second wave of TUNEL-positive cells and significant optic nerve degeneration at 28 days post-blast wave exposure. The detection of TUNEL-positive cells prior to any degenerating axons in the optic nerve suggests that the axon degeneration is secondary to RGC death.

## Conclusions

In summary, this study demonstrates that a blast overpressure airwave directed at the eyes of young DBA/2 J mice can be used as a model for open globe ocular trauma. This model is attractive because the globe remains intact, avoiding complications from potential bacterial infections. Treatment options for open globe trauma patients are very limited and none have shown a high degree of success. We expect that this model will be useful as a platform for identifying the mechanisms underlying ongoing vision loss and testing potential therapeutics for the treatment of open globe ocular trauma.

## Additional files

Additional file 1: Figure S1. Treatment with non-medicated eye drops after blast reduces RPE damage. **(A-B)** Representative light micrographs of non-eye drop treated RPE **(A)** and eye drop treated RPE **(B)** at 3 days post-injury. The scale bar in **(A)** is 25 μm and applies to both images. RPE = retinal pigment epithelium. Additional file 2: Figure S2. Non-medicated eye drop treatment reduces immune infiltrate. **(A-B)** Representative light micrographs of cornea **(A)** and the ciliary body **(B)** from an eye drop treated eye at 3 days post-injury. The scale bar in **(A)** is 25 μm and applies to both images. Additional file 3: Figure S3. Nitrotyrosine immunolabeling is more focal after eye drop treatment. **(A-B)** Representative low and high magnification epifluorescence micrographs of a non-eye drop treated retina **(A)** and an eye drop treated retina **(B)** at 3 days post-injury. The scale bar in the low magnification image **(A)** is 250 μm and applies to both low magnification images. The scale bar in the high magnification image **(A)** is 50 μm and applies to both high magnification images. White boxes in the low magnification images correspond to the high magnification images. Additional file 4: Figure S4. Microglial reactivity is limited after eye drop treatment. **(A-B)** Representative low and high magnification epifluorescence micrographs of a non-eye drop treated retina **(A)** and an eye drop treated retina **(B)** at 3 days post-injury. The white boxes denote reactive microglia and the arrows point to the corresponding high magnification images. The scale bar in the low magnification image **(A)** is 250 μm and applies to both low magnification images. The scale bar in the high magnification image **(A)** is 50 μm and applies to both high magnification images.

## Abbreviations

ACAID: Anterior chamber-associated immune deviation
DAPI: 4’,6-diamidino-2-phenylindole
Endo: Endothelium
Epi: Epithelium
ERG: Electroretinogram
C3d: Complement 3d
CA: Corneal abrasions
CE: Corneal edema
CNV: Corneal neovascularization
GCL: Ganglion cell layer
GFAP: Glial fibrillary acidic protein
IBA-1: Ionized calcium binding adaptor molecule 1
INL: Inner nuclear layer
OCT: Optical coherence tomography
OKN: Optokinetic nystagmus
ONH: Optic nerve head
ONL: Outer nuclear layer
PBS: Phosphate buffered saline
PBT: Phosphate buffer with bovine serum albumin and triton X 100
PFA: Paraformaldehyde
PMD: Pupilomotor deficit
PVC: Polyvinylchloride
RGC: Retinal ganglion cell
RIP1: Receptor interacting protein 1
RIP3: Receptor interacting protein 3
RPE: Retinal pigment epithelium
TUNEL: TdT dUTP nick end labeling
