# Erratum to: Exacerbation of blast-induced ocular trauma by an immune response

**DOI:** 10.1186/s12974-016-0704-6

**Published:** 2016-08-30

**Authors:** Courtney Bricker-Anthony, Jessica Hines-Beard, Lauren D’Surney, Tonia S. Rex

**Affiliations:** 1 Vanderbilt Eye Institute, Vanderbilt University, 11425 MRB IV, 2213 Garland Ave., Nashville, TN 37232 USA; 2 Vanderbilt Brain Institute, Vanderbilt University, 11425 MRB IV, 2213 Garland Ave., Nashville, TN 37232 USA; 3 Department of Ophthalmology, University of Tennessee Health Science Center, 930 Madison Ave., Memphis, TN 38103 USA

## Erratum

Upon publication of the original article [1], it was noticed that the software on the Cerebral Mechanics OptoMotry system was mis-interpreted. It states “CW (right)” and “CCW (left)”, thus the individual performing the assay understood “right” to mean “right eye” and “left” to mean “left eye”, when in fact the use of the terms “right” and “left” in the software refer to the direction of the motion of the contrasting bars. The eyes that are being tested are actually the opposite. Thus the graph in Fig. 12d (and additional file 12, which has the Figure in it too) of the paper is of the contralateral rather than the ipsilateral eye to the over-pressure air-wave. Please see the below correct graph. This does not change the interpretation of the effect of this injury on the eye. The only difference is the lack of a statistically significant decrease at 14 days after injury.

**Fig. 12:**
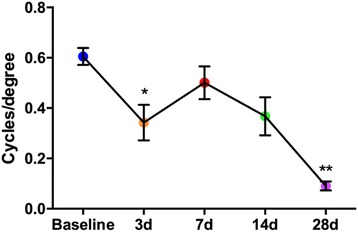
Blast wave exposure causes early and late visual deficits; **d** Photopic spatial threshold (visual acuity) is significantly decreased at three, 14 and 28 days post-blast wave exposure. *P <0.05, **P <0.01. Error bars represent SEM for each graph
