# Supplementary figures and images for: Exacerbation of blast-induced ocular trauma by an immune response
Source: J Neuroinflammation. 2014 Nov 29;11(1):192. doi: 10.1186/s12974-014-0192-5 (PMC4264554; doi:10.1186/s12974-014-0192-5)

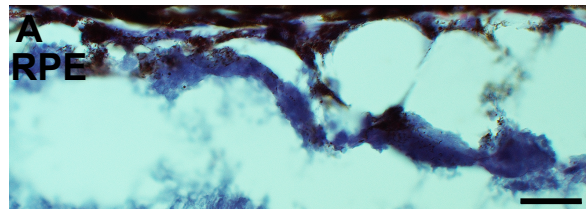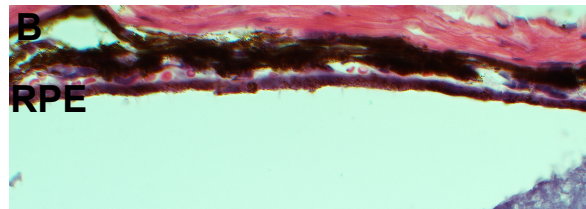

Supplement: Additional file 1: Figure S1. — Treatment with non-medicated eye drops after blast reduces RPE damage. (A-B) Representative light micrographs of non-eye drop treated RPE (A) and eye drop treated RPE (B) at 3 days post-injury. The scale bar in (A) is 25 μm and applies to both images. RPE = retinal pigment epithelium. [file 12974_2014_192_MOESM1_ESM.pdf]

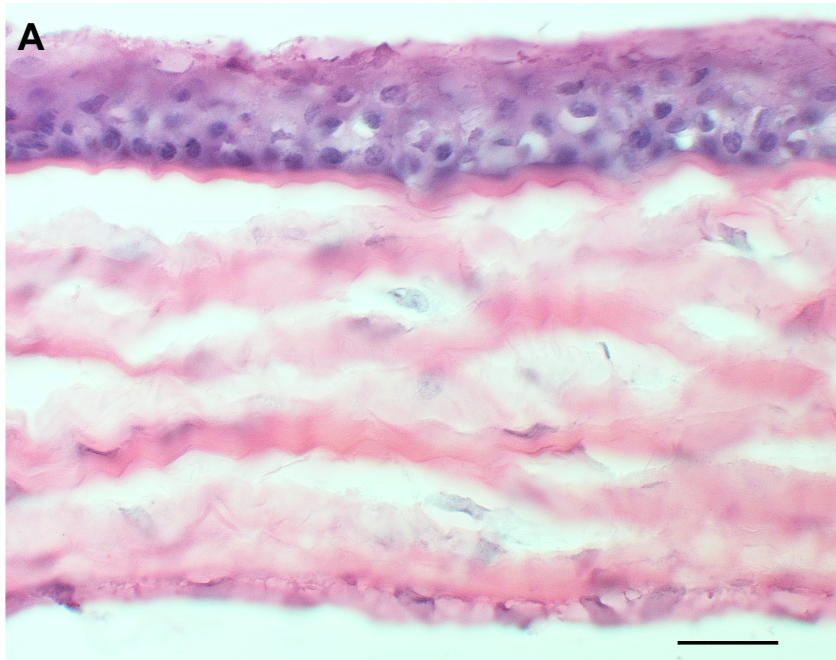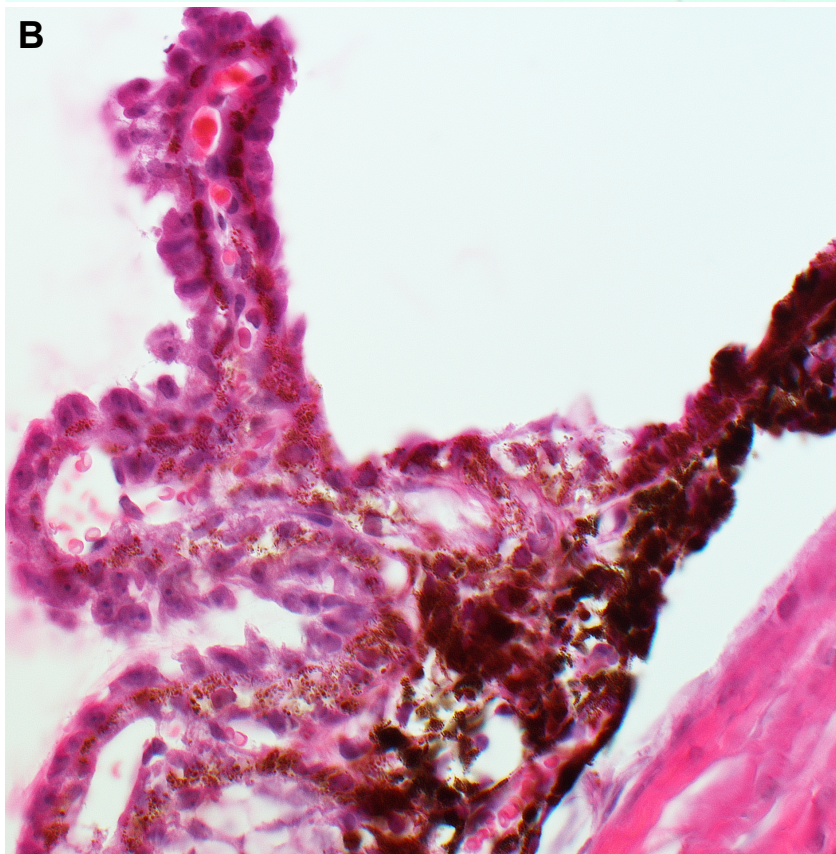

Supplement: Additional file 2: Figure S2. — Non-medicated eye drop treatment reduces immune infiltrate. (A-B) Representative light micrographs of cornea (A) and the ciliary body (B) from an eye drop treated eye at 3 days post-injury. The scale bar in (A) is 25 μm and applies to both images. [file 12974_2014_192_MOESM2_ESM.pdf]

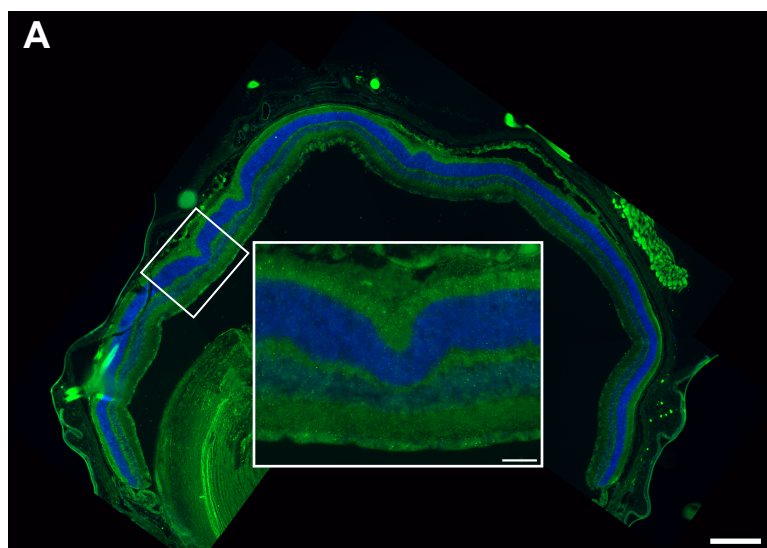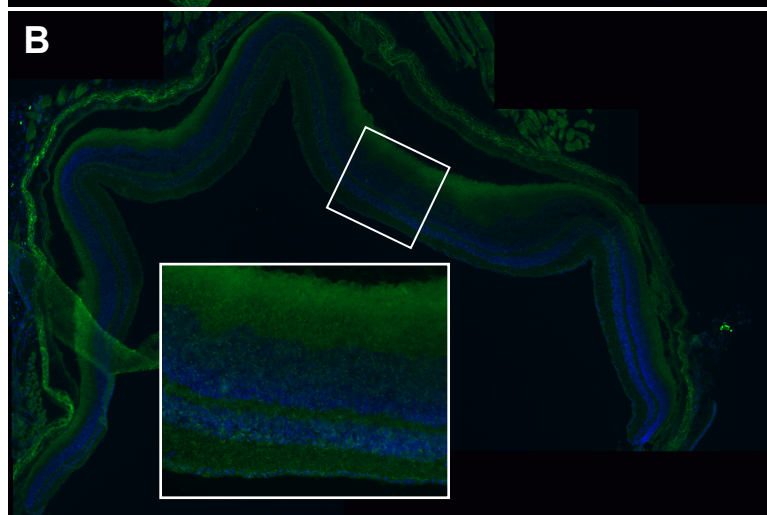

Supplement: Additional file 3: Figure S3. — Nitrotyrosine immunolabeling is more focal after eye drop treatment. (A-B) Representative low and high magnification epifluorescence micrographs of a non-eye drop treated retina (A) and an eye drop treated retina (B) at 3 days post-injury. The scale bar in the low magnification image (A) is 250 μm and applies to both low magnification images. The scale bar in the high magnification image (A) is 50 μm and applies to both high magnification images. White boxes in the low magnification images correspond to the high magnification images. [file 12974_2014_192_MOESM3_ESM.pdf]

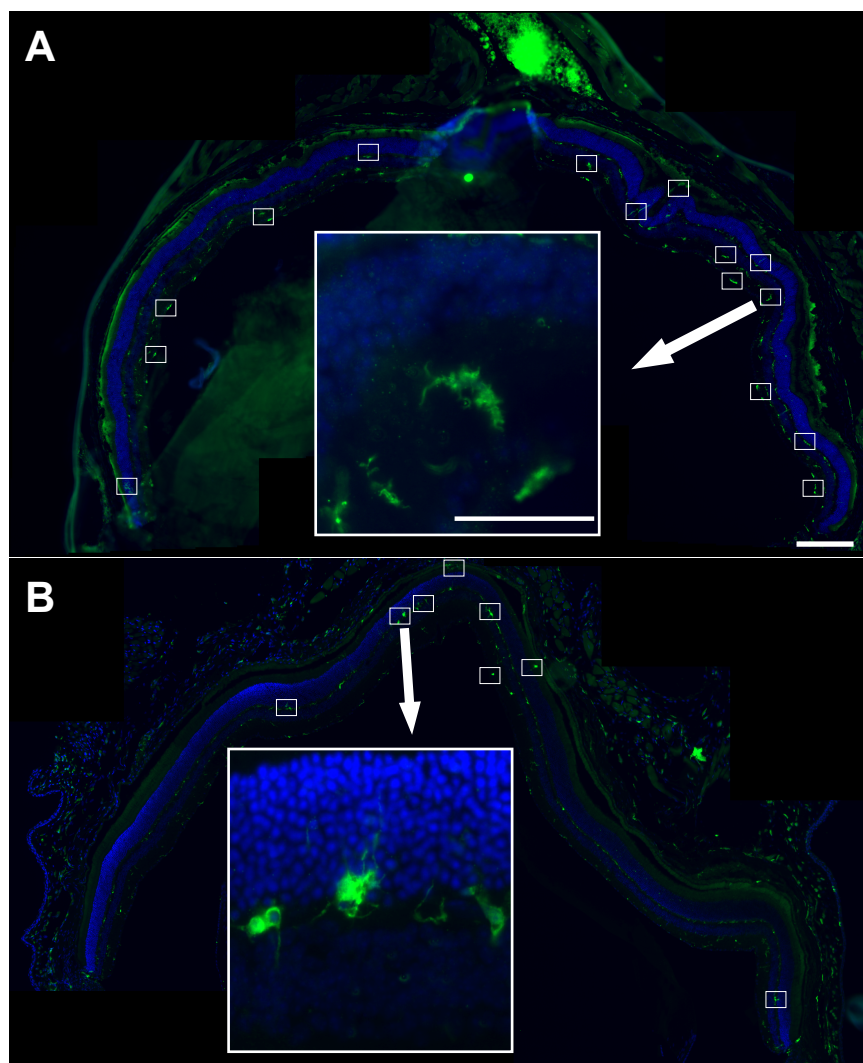

Supplement: Additional file 4: Figure S4. — Microglial reactivity is limited after eye drop treatment. (A-B) Representative low and high magnification epifluorescence micrographs of a non-eye drop treated retina (A) and an eye drop treated retina (B) at 3 days post-injury. The white boxes denote reactive microglia and the arrows point to the corresponding high magnification images. The scale bar in the low magnification image (A) is 250 μm and applies to both low magnification images. The scale bar in the high magnification image (A) is 50 μm and applies to both high magnification images. [file 12974_2014_192_MOESM4_ESM.pdf]
